# Welche Potenziale und Mehrwerte bieten DiGA für die hausärztliche Versorgung? – Ergebnisse einer Befragung von Hausärzt*innen in Deutschland

**DOI:** 10.1007/s00103-022-03608-w

**Published:** 2022-10-21

**Authors:** Julian Wangler, Michael Jansky

**Affiliations:** Zentrum für Allgemeinmedizin und Geriatrie, Universitätsmedizin Mainz, Am Pulverturm 13, 55131 Mainz, Deutschland

**Keywords:** Digitale Gesundheitsanwendung, DiGA, Gesundheits-Apps, M‑Health, Prävention, Gesundheitsförderung, Hausarzt, Digital health applications, DiGA, Health apps, MHealth, Prevention, Health promotion, General practitioner

## Abstract

**Hintergrund:**

Für Ärzt*innen besteht die Möglichkeit, Patient*innen digitale Gesundheitsanwendungen (DiGA) auf Rezept zu verordnen. Bislang fehlen Untersuchungen, die Anwendungsmöglichkeiten von DiGA im hausärztlichen Praxiskontext beleuchten und eine erste Bilanz zum Nutzen sowie Optimierungspotenzial ziehen.

**Ziel der Arbeit:**

Die Studie exploriert hausärztliche Einstellungen, Erwartungen und Erfahrungswerte mit Blick auf die Anwendungspotenziale von DiGA.

**Methoden:**

Im Frühjahr 2022 wurden sämtliche 13.913 als Behandler*innen aktive Hausärzt*innen in Baden-Württemberg, Hessen, Rheinland-Pfalz und im Saarland zu einer Online-Befragung eingeladen. 3829 vollständig ausgefüllte Fragebögen gingen in die Auswertung ein (Rücklauf: 28 %). Zur Ermittlung von signifikanten Unterschieden zwischen 2 Gruppen erfolgten ein t‑Test bei unabhängigen Stichproben sowie eine Faktorenanalyse.

**Ergebnisse:**

Die meisten Befragten erachten DiGA als verlässliche (67 %) und sichere (61 %) Anwendungen. 22 % trauen sich zu, Patient*innen zu DiGA kompetent zu beraten. 14 % haben bereits DiGA verschrieben, 13 % haben dies vor. 83 % der Ärzt*innen mit DiGA-Erfahrung bewerten die verordneten Anwendungen als nützlich. Beobachtete Versorgungseffekte betreffen v. a. die Verbesserung von Therapietreue (95 %), Mobilität (94 %) und Aufklärung (93 %) sowie Gewichtsreduktion (82 %). Angeregt wird u. a. eine weitere Optimierung der Nutzerfreundlichkeit (59 %), die systematische Weiterbildung von Ärzt*innen (52 %) und die verstärkte Aufnahme spielerischer Elemente (49 %).

**Diskussion:**

Hausärzt*innen sollten besser über die Grundlagen des Digitale-Versorgung-Gesetzes (DVG) informiert und Bedenken gezielt adressiert werden. Zentral erscheinen flächendeckende Schulungen, die über Rahmenbedingungen und Vorteile des DiGA-Einsatzes aufklären. Auch besteht Bedarf an fundierteren Informations- und Recherchequellen für Ärzt*innen.

**Zusatzmaterial online:**

Zusätzliche Informationen sind in der Online-Version dieses Artikels (10.1007/s00103-022-03608-w) enthalten.

## Abstract (en)

**Background:**

For almost two years, physicians have been able to prescribe digital health applications (DiGA) to patients. So far, there has been a lack of studies that shed light on the possible use of DiGA in the context of primary care and take stock of the benefits and potential for optimization.

**Objectives:**

This study explores general practitioners’ (GPs) attitudes, expectations, and experiences with regard to the application potential of DiGA.

**Methods:**

In the spring of 2022, all 13,913 general practitioners in the federal states Baden-Württemberg, Hesse, Rhineland-Palatinate, and Saarland were invited to take part in an online survey. Included in the evaluation were 3829 completed questionnaires (response rate: 28%). A t-test on independent samples was used to determine significant differences between two groups. Moreover, a factor analysis was carried out.

**Results:**

A majority of those surveyed consider DiGA to be reliable (67%) and secure (61%) applications. In addition, 22% trust themselves to competently advise patients on DiGA, 14% have already prescribed DiGA, and 13% plan to do so. Of the GPs with DiGA experience, 83% rate the prescribed applications as useful. Observed healthcare effects mainly relate to the improvement of compliance (95%), mobility (94%), and education (93%) as well as weight reduction (82%). The respondents suggest further optimization of DIGA, especially with regard to usability (59%), systematic further training of doctors (52%), and the expansion of gamification elements (49%).

**Conclusions:**

It is important to better inform GPs about the basics of the Digital Healthcare Act (DVG) and to address concerns in a targeted manner. Comprehensive training courses that explain the framework conditions and advantages of using DiGA appear to be crucial. There is also a need for more well-founded sources of information and research for doctors.

## Einleitung

Als Teil einer umfassenden Digitalstrategie hat der deutsche Gesetzgeber mit dem Digitale-Versorgung-Gesetz (DVG) vom 19.12.2019 die Möglichkeit geschaffen, digitale Gesundheitsanwendungen (DiGA) in die Regelversorgung zu integrieren [1]. Seitdem besteht für Ärzt*innen die Möglichkeit, gesetzlich versicherten Patient*innen DiGA auf Rezept zu verordnen. Als erstattungsfähige mobile Apps sollen DiGA bei der effektiveren Erkennung von Erkrankungen helfen, Therapien begleiten und/oder zur Prävention beitragen [2]. Im Gegensatz zu konventionellen Gesundheits-Apps gelten DiGA als Medizinprodukte. Ihnen wurde eine niedrige Risikoklasse zugewiesen [3].

Damit eine DiGA als erstattungsfähig eingestuft wird, bedarf es einer Aufnahme in das vom Bundesinstitut für Arzneimittel und Medizinprodukte (BfArM) geführte DiGA-Verzeichnis [4]. Hierzu müssen DiGA-Hersteller einen Antrag auf Zulassung stellen und in einem systematischen Evaluationsverfahren verschiedene Anforderungen erfüllen. So ist es neben einer CE-Kennzeichnung als Medizinprodukt erforderlich, dass Standards zu Datenschutz und Informationssicherheit, Qualität medizinischer Inhalte, Nutzerfreundlichkeit (Usability) und Robustheit der Anwendung sowie Patient*innensicherheit eingehalten werden. Nutzen und Mehrwert der Anwendung (Versorgungseffekt) müssen ausreichend belegt werden [5]. Bei Erfüllung aller Beurteilungskriterien sind eine Aufnahme ins DiGA-Verzeichnis und ergo eine Verordnung möglich. Sollten zunächst lediglich die allgemeinen Anforderungen erfüllt sein, besteht die Option auf vorübergehende Aufnahme über das sogenannte Fast-Track-Verfahren. Hier hat der Hersteller im Zuge einer einjährigen Erprobungsphase Zeit, einen Nachweis über die positiven Versorgungseffekte vorzulegen [2, 3]. Dabei können Versorgungseffekte in unterschiedlichen Kategorien belegt werden (z. B. Rückgang von Schmerzen und/oder größeres Wissen bzw. besserer Umgang in Bezug auf eine Erkrankung). DiGA sollten so ausgelegt sein, dass sie von Patient*innen allein oder gemeinsam mit Ärzt*innen verwendet werden können [2, 6].

Inzwischen liegen zu immer mehr Krankheitsbildern (z. B. Migräne, Tinnitus, Adipositas, Diabetes, Schlafstörungen, psychische Erkrankungen) positiv evaluierte Programme vor. Von den 35 DiGA, die mit Stand vom September 2022 beim BfArM gelistet sind, ist das Gebiet der Lebensstil-assoziierten Anwendungen vergleichsweise stark vertreten. Studien konnten nachweisen, dass Gesundheits-Apps positive Effekte bei Erkrankungen wie Adipositas oder Diabetes mellitus Typ 2 haben können, indem eine alltagsregulierende Veränderung des Patient*innenverhaltens (z. B. Ernährung, Bewegung) induziert wird [7–11].

Ähnlich wie konventionelle Gesundheits-Apps werden DiGA v. a. mit dem Potenzial in Verbindung gebracht, Patient*innen bei der Bewältigung von Erkrankungen dahingehend zu unterstützen, dass Eigenverantwortung (Empowerment), intrinsische Motivation und Therapietreue (Compliance) profitieren. Durch edukative Inhalte kann die Aufklärung verbessert und mittels Erinnerungsfunktionen gesundheitsförderliches Verhalten eingeübt werden [2, 3, 6, 8]. Idealerweise lassen sich Krankheitsrisiken unter Einbindung einer App früher identifizieren, Problematiken und Symptome dokumentieren und das Arzt-Patient-Verhältnis effektiver gestalten [12].

Für die erfolgreiche Etablierung von DiGA im Gesundheitswesen nehmen Hausärzt*innen in der Rolle von Primärversorger*innen eine Schlüsselstellung ein [13, 14]. So ist vorstellbar, dass Hausärzt*innen DiGA gezielt zur Gesundheitsförderung einsetzen (z. B. zur kardiovaskulären Risikoprävention), den Anwendungsprozess begleiten und von Patient*innen regelmäßig Vitaldaten erhalten [15, 16]. Expertisen heben den Nutzen von Gesundheits-Apps als Hilfsmittel bei der Optimierung von Differenzialdiagnostik, Krankheitsmanagement und Therapietreue hervor [2, 3, 7, 8].

Erhebungen haben gezeigt, dass gerade niedergelassene Ärzt*innen zwar Einsatzpotenziale von Gesundheits-Apps ausmachen (u. a. Stärkung der Eigenverantwortung, Aufklärung, Arzt-Patient-Vernetzung), bislang allerdings erkennbar zurückhaltend waren, solche zu empfehlen und in die Versorgung zu integrieren [13, 17–19]. Diese Zurückhaltung korrespondiert mit einer ausgeprägten Unsicherheit hinsichtlich der Zuverlässigkeit und Sicherheit (Datenschutz, Praxisreife, Eignung für bestimmte Patient*innenklientelen, Rechtssicherheit bei ärztlich induziertem App-Einsatz) sowie der Integration in den Versorgungsalltag [19–21]. Solchen Bedenken trägt das DiGA-Konzept Rechnung. Angesichts des schwer überschaubaren App-Marktes artikulieren die meisten Ärzt*innen einen schlechten Informationsstand und Schwierigkeiten, geeignete Gesundheits-Apps auszuwählen [21].

Bislang fehlen belastbare Untersuchungen, die Anwendungsmöglichkeiten speziell von DiGA im hausärztlichen Praxiskontext beleuchten. Vor dem Hintergrund der im Jahr 2020 erfolgten Etablierung solcher M‑Health-Tools (mobile Werkzeuge für Gesundheit) im deutschen Gesundheitswesen ermittelt die vorliegende Studie eine Zwischenbilanz aus allgemeinärztlicher Perspektive. Dabei werden nicht nur Kenntnisstand, Einstellungen (Akzeptanz, Chancen und Risiken, Anwendungspotenziale nach Bereichen), Erwartungen, Anwendungsbereitschaft und Erfahrungswerte von Hausärzt*innen mit Blick auf DiGA erfasst. Auch soll eruiert werden, inwiefern Hausärzt*innen in DiGA gegenüber der Anwendung konventioneller Gesundheits-Apps einen Mehrwert ausmachen. Aus den Ergebnissen sollen Rückschlüsse gezogen werden, unter welchen Voraussetzungen die Potenziale von DiGA für die (primär-)ärztliche Versorgung nutzbar gemacht werden können.

## Methodik

Zur Adressierung des Forschungsdesiderats hat die explorative Untersuchung ein ausführliches Meinungs- und Erfahrungsbild unter Hausärzt*innen eingeholt. Hierzu erfolgte im Frühjahr 2022 eine Vollbefragung von Hausärzt*innen in 4 Bundesländern. Diese war ausgestaltet als Onlinebefragung mit postalischem Anschreiben.

### Erhebungsinstrument

Die Konzeption des Befragungsinstruments (siehe Onlinezusatzmaterial) erfolgte unter Rückgriff auf mehrere quantitative und qualitative Vorstudien der Autoren, die sich unter verschiedenen Fokussen mit den Einsatzmöglichkeiten von Gesundheits-Apps im haus- und fachärztlichen Setting befasst haben [19, 20, 22], sowie einer Literaturrecherche (u. a. [6–8, 12, 15, 17, 18, 21, 23, 24]). Es besteht aus 27 Fragen mit 4 inhaltlichen Schwerpunkten:

- Bekanntheit und generelle Beurteilung des DiGA-Konzepts,
- Einstellungen und wahrgenommene Nutzungspotenziale von DiGA für die Versorgung,
- Voraussetzungen einer DiGA-Anwendung im Praxiskontext,
- Anwendungsbereitschaft und -erfahrung in Bezug auf DiGA.

Die Ergebnisse der Vorstudien flossen v. a. bei der Erstellung der verwendeten Itembatterien ein (Frage 6, 7, 12, 14, 17).

Neben den standardisierten Fragen wurden mehrere offene Fragen eingesetzt (Frage 13, 15, 18, 24).

Um bei der zeitlich stark ausgelasteten Zielgruppe der Hausärzt*innen einen guten Kompromiss aus Datenqualität und intuitiver Beantwortbarkeit des Fragebogens zu erreichen, wurden vermehrt Ordinalskalen eingesetzt. Diese waren normalerweise 4‑stufig; im Fall einiger Ausnahmen (Frage 6, 12, 17) erschien es vertretbar, die beiden negativen Antwortoptionen zusammenzufassen. Auf die Verwendung einer neutralen Mittelkategorie bei der Einstufung wurde bewusst verzichtet, da Tendenzen bei der Einstellung und Einschätzung in Bezug auf DiGA möglichst deutlich werden und das Ankreuzen von Mittelkategorien im Sinne einer Non-Response vermieden werden sollten. Hingegen wurden bei verschiedenen Wissens- und Einschätzungsfragen ergänzende Antwortmöglichkeiten verwendet, bei denen die Befragten markieren konnten, dass ihnen eine Einschätzung schwerfällt.

Als soziodemografische Merkmale wurden Geschlecht, Alter, Praxisumgebung, Praxisform und Anzahl der Patient*innen pro Quartal erhoben. Vor dem Feldeinsatz wurde ein Pretest durchgeführt. Hierzu wurde der Fragebogen 50 zufällig ausgewählten Hausärzt*innen aus dem Umfeld der hausärztlichen Lehrbeauftragten der Abteilung Allgemeinmedizin vorgelegt. Der Pretest zeigte, dass eine gute Verständlichkeit und Strukturierung sowie Vollständigkeit der Antwortkategorien gegeben sind. Kleinere Itemergänzungen erfolgten bei 2 Itembatterien (Frage 7, 14).

### Rekrutierung und Stichprobe

Auf schriftlich-postalischem Weg zur Teilnahme an der anonymisierten Befragung eingeladen wurden zwischen März und Mai 2022 sämtliche 13.913 als Behandler*innen aktive Hausärzt*innen in Baden-Württemberg (6664), Hessen (3839), Rheinland-Pfalz (2667) und im Saarland (743). Es handelte sich um ein einmaliges Anschreiben, in dem die zu befragenden Ärzt*innen u. a. einen passwortgeschützten Zugang zur Onlinebefragung mitgeteilt bekamen (keine Incentives).

Von den 3845 bearbeiteten Fragebögen gingen 3829 vollständig ausgefüllte Bögen in die Auswertung ein (Rücklauf: 28 %). Tab. 1 vergleicht die Merkmale der gewonnenen Stichprobe mit den Merkmalen der allgemeinen Hausärzteschaft, wie sie aus repräsentativen Versorgungsforschungsdaten der Kassenärztlichen Vereinigungen (KV) für Rheinland-Pfalz bzw. Deutschland hervorgehen.

**Table 1:** 

	Stichprobe (*N* = 3829)	Repräsentativstatistik
Geschlecht:	60 % männlich, 40 % weiblich	58 % männlich, 42 % weiblich^a^
Durchschnittsalter:	54 (Median: 54)	56 (Median: 57)^a^
Praxisumgebung:	58 % mittel- und großstädtisch, 42 % ländlich-kleinstädtisch	41 % mittel- und großstädtisch, 59 % ländlich-kleinstädtisch^a^
Praxisform:	53 % Einzelpraxen, 33 % Gemeinschaftspraxen, 14 % MVZ/sonstige Einrichtung	56 % Einzelpraxen, 38 % Gemeinschaftspraxen, 6 % MVZ/sonstige Einrichtung^b^
Patienten pro Quartal:	17 % 500–1500, 36 % 1501–2000, 47 % > 2000	Keine vollständigen Daten verfügbar

*MVZ* medizinisches Versorgungszentrum

^a^Basierend auf den KV-Versorgungsforschungsdaten für Rheinland-Pfalz (Stand: 31.12.2020), abrufbar unter: https://www.kv-rlp.de/institution/engagement/versorgungsforschung/

^b^Basierend auf den KV-Versorgungsforschungsdaten für Deutschland (Stand: 31.12.2020), abrufbar unter: https://gesundheitsdaten.kbv.de/

### Datenanalyse

Die Daten wurden mittels SPSS 23.0 ausgewertet. Zur Ermittlung von signifikanten Unterschieden zwischen 2 Gruppen erfolgte ein t‑Test bei unabhängigen Stichproben. Zwecks Gruppenvergleich herangezogen wurde u. a. die Praxisumgebung, wobei hier die Kategorien groß- bzw. mittelstädtisch und kleinstädtisch-ländlich gegenübergestellt wurden. Mit Blick auf das Alter wurde zwischen Befragten ober- und unterhalb des Durchschnittsalters unterschieden.

Zudem wurde eine Faktorenanalyse (Varimax-Rotation) durchgeführt. Die Faktorenanalyse dient dazu, eine größere Zahl von Variablen aufgrund von systematischen Beziehungen (Korrelationen) untereinander zu Faktoren zusammenzufassen. Hierdurch sollen zugrunde liegende gemeinsame Dimensionen aufgedeckt werden. Die gewählte Varimax-Methode ist das geläufigste Verfahren, um zu interpretierbaren Faktorenlösungen zu gelangen. Als Grenze, ab der ein Item auf einen Faktor lädt, wurden die Werte 0,4/−0,4 gewählt [25]. Um die Voraussetzung für eine Faktorenanalyse zu prüfen, wurde der Bartlett-Test auf Sphärizität durchgeführt. Dieser prüft die Hypothese, dass in der Grundgesamtheit alle Korrelationskoeffizienten den Wert Null haben. Ein signifikantes Ergebnis erlaubt die Interpretation, dass in der Grundgesamtheit zumindest zwischen einigen Variablen Korrelationen bestehen; die Nullhypothese kann zurückgewiesen werden.

Die Auswertung der offenen Fragen basiert auf einer Nachcodierung im Sinne der qualitativen Inhaltsanalyse. Dies beinhaltete für die Freitextantworten zu jeder offenen Frage die Erstellung eines basalen Kategoriensystems [26]. Als Reporting Statement wurde STROBE herangezogen.

## Ergebnisse

### Bekanntheit und Beurteilung des DiGA-Konzepts

87 % der Befragten ist zum Befragungszeitpunkt bekannt gewesen, dass für Ärzt*innen die Möglichkeit besteht, DiGA auf Rezept zu verordnen. In Groß- und Mittelstädten ansässige Ärzt*innen sind zu 95 % über DiGA informiert, während dies unter Ärzt*innen in Kleinstädten und Landgemeinden 71 % sind (*p* < 0,001). 67 % der Befragten stehen DiGA grundsätzlich positiv gegenüber, wohingegen sich 17 % skeptisch zeigen (16 % unentschieden). Ärzt*innen in urbanen Umgebungen nehmen Apps erheblich positiver als ihre Kolleg*innen auf dem Land wahr (94 % zu 27 %, *p* < 0,001). Ebenso beurteilen Befragte unterhalb des Durchschnittsalters solche Anwendungen klar positiver als ältere Ärzt*innen (85 % zu 49 %, *p* < 0,001).

67 % der Befragten bekunden grundsätzlich Vertrauen in das Evaluationsverfahren des BfArM zur Zulassung von DiGA und gehen davon aus, dass es sich bei gelisteten DiGA um verlässliche Anwendungen handelt. Während 93 % der städtischen Ärzt*innen Vertrauen in das DiGA-Verzeichnis bekunden, fällt dieses bei Landärzt*innen mit 28 % beträchtlich niedriger aus (*p* < 0,001). 61 % aller Befragten glauben, dass es bei der Verschreibung von DiGA und deren Nutzung im Praxiskontext ausreichend Rechtssicherheit für Ärzt*innen gibt (z. B. mit Blick auf Fragen zu Risiken und Haftung).

29 % der Befragten gehen davon aus, dass DiGA einen sehr großen oder eher großen Beitrag zur Gesundheitsförderung leisten können; 61 % halten den Beitrag für vorhanden, aber eher gering (10 % kein Beitrag). 38 % der urbanen Ärzt*innen gehen von einem großen Beitrag durch DiGA aus gegenüber 9 % der Landärzt*innen (*p* < 0,001). Bezogen auf sämtliche Befragte nehmen 58 % an, dass DiGA die Digitalisierung von Gesundheitswesen und Arztpraxen in positiver und sinnvoller Weise voranbringen werden (16 % nein, 26 % schwer zu sagen).

### Einstellungen und Nutzungspotenziale in Bezug auf DiGA

Der wahrgenommene Nutzen von DiGA variiert nach Anwendungsfeld. 86 % halten es für sinnvoll, wenn diese beim Management von Medikamenten oder ärztlichen Terminen helfen. 74 % sehen die Unterstützung bei der Selbstkontrolle von Risikofaktoren (Gewicht, Blutdruck, Blutzucker etc.) oder Gewichtsdaten (gelaufene Schritte, Trinkmenge etc.) positiv. 72 % befürworten den Einsatz von DiGA bei physischen Maßnahmen, gefolgt von 70 % für Funktionen, die bei der Einhaltung eines gesunden Lebensstils (z. B. Ernährung, Rauchentwöhnung) helfen sollen. Für das Monitoring und die Therapieunterstützung sehen 53 % DiGA als gut geeignet an.

54 % der Befragten halten es grundsätzlich für in Ordnung, wenn Ärzt*innen sich bei der Therapieplanung auf Daten verlassen, die von Patient*innen mittels DiGA erfasst wurden, während 27 % ablehnend antworten (19 % schwer zu sagen). Unter denjenigen Befragten, die dies für in Ordnung halten, beträgt der Anteil, der DiGA zur Unterstützung von Krankheitsmanagement und Therapie befürwortet, 69 %.

Die Befragten bringen DiGA mit Chancen und Risiken in Verbindung (Tab. 2). So wird eine Motivations- und Therapietreuesteigerung als wesentlicher Vorzug erachtet. Auch die Dimension einer Stärkung des Empowerments wird als bedeutsam eingeschätzt. Zugleich bringt ein Teil der Befragten Sorgen vor mangelnder (Daten‑)Sicherheit zum Ausdruck und befürchtet unerwünschte Effekte wie Fehlmessungen aufgrund einer zu geringen Eignung für bestimmte Patient*innengruppen.

**Table 2:** 

		Rotierte Komponentenmatrix		
**Frage:** *Welchen der folgenden Aussagen stimmen Sie tendenziell zu?* (*N* = 3829)	Gesamtzustimmung (%)	Komponente 1 (Varianzaufklärung: 18,8 %)	Komponente 2 (Varianzaufklärung: 17,8 %)	Komponente 3 (Varianzaufklärung 12,4 %)
DiGA stärken die Motivation von Patient*innen, etwas für ihre Gesundheit zu tun	65	0,739	0,237	0,071
DiGA stärken die Compliance von Patient*innen	51	0,766	0,141	0,214
Ich glaube nicht, dass DiGA personenbezogene Daten ausreichend schützen	44	−0,120	−0,178	0,745
DiGA geben Patient*innen das Gefühl, stärkere Kontrolle über ihre Gesundheit und Gesunderhaltung zu haben (Empowerment)	35	0,403	0,194	0,113
Die Anwendung von DiGA ist für viele Patient*innen zu kompliziert, wodurch fehlerhafte Gesundheitsdaten gesammelt werden oder im Extremfall Therapien fehlschlagen	35	0,398	−0,667	0,226
DiGA tragen dazu bei, dass Patient*innen besser über Gesundheits- und Krankheitsthemen aufgeklärt sind	29	0,729	0,128	−0,292
Durch DiGA lassen sich Arztkontakte zeitlich effektiver gestalten	27	0,361	0,792	0,122
Durch DiGA wird das Arzt-Patient-Verhältnis unpersönlicher	23	−0,480	−0,210	0,019
Krankheiten und Krankheitsrisiken können aufgrund der Nutzung von DiGA schneller erkannt bzw. diagnostiziert werden	22	0,326	0,824	−0,100
DiGA entlasten Ärzt*innen und Pflegekräfte, weil diese sich nicht mehr um die Erfassung von Vitaldaten und Messwerten kümmern müssen	21	0,626	0,104	−0,314
Durch den Einsatz von DiGA beim Patienten wird der Arzt stärker belastet als entlastet, weil zusätzliche Aufgaben für ihn anfallen	20	0,271	−0,331	−0,600
Mit DiGA lassen sich neue Patient*innengruppen erreichen	19	−0,026	−0,400	−0,042
DiGA erzeugen eine Flut von Daten, die einer effektiven Behandlung nicht zweckdienlich sind	18	0,105	−0,282	0,733
Die Nutzung von DiGA ist für Patient*innen und Ärzt*innen zu zeitaufwändig	11	−0,410	−0,325	0,059
DiGA erleichtern die Kommunikation zwischen Arzt und Patient	11	0,325	0,718	0,025
Durch die zusätzlichen Informationen, die von DiGA erfasst werden, können Patient*innen individueller und effektiver behandelt werden	6	0,257	0,554	−0,206
DiGA führen dazu, dass Patient*innen ihre Diagnose und Therapie in die eigene Hand nehmen	3	−0,163	−0,123	0,044

Extraktionsmethode: Hauptkomponentenanalyse

Rotationsmethode: Varimax, Kaiser-Normalisierung

Rotation in 7 Iterationen konvergiert

Aufgeklärte Gesamtvarianz: 49,0 %

Stichprobeneignung nach Kaiser-Meyer-Olkin: 0,666

Signifikanz nach Bartlett: *p* < 0,001

Kommunalitäten aller eingeschlossenen Variablen über Grenzwert 0,5

Eine Faktorenanalyse, bei der Variablen aufgrund systematischer Beziehungen (Korrelationen) untereinander zu Faktoren zusammengefasst werden [25], zeigt 3 Cluster von Hausärzt*innen. Innerhalb der ersten Gruppe korrelieren wahrgenommene Vorteile von DiGA für die Gesundheitsförderung, Therapietreue und Motivation. In der zweiten Gruppe wird v. a. auf Effektivitäts- und Effizienzvorteile für die Arzt-Patient-Vernetzung abgehoben. Zugleich haben die Befragten Gefahren einer Überbeanspruchung sowie einer Fehlbehandlung aufgrund des App-Einsatzes vor Augen. Im dritten Cluster sind negative Einstellungen anzutreffen, die sich auf Aspekte wie Datenschutz und negative Konsequenzen für die Arzt-Patient-Beziehung beziehen.

### Voraussetzungen einer DiGA-Anwendung

Digitale Gesundheitsprogramme sind in der Alltagsrealität der Patient*innen inzwischen verbreitet. 42 % der Befragten schätzen, dass zwischen 10 % und 15 % der eigenen Patient*innen regelmäßig Gesundheits-Apps und/oder andere digitale Tools zur gesundheitlichen Vorsorge bzw. zum Krankheitsmanagement nutzen; weitere 38 % vermuten eine Nutzerverbreitung zwischen 15 % und 20 %. Im Durchschnitt gehen die Ärzt*innen davon aus, dass die Nutzung von DiGA für 15 % der eigenen Patient*innen interessant wäre bzw. ist. Unter Ärzt*innen in Großstädten beträgt der Anteil 23 %.

18 % der Befragten geben an, gelegentlich von den eigenen Patient*innen auf DiGA angesprochen zu werden; bei 42 % kommt dies höchstens vereinzelt vor (41 % nie). Ärzt*innen, die in Bezug auf DiGA bereits angesprochen wurden, finden sich meist im mittel- bis großstädtischen Raum, deutlich seltener in Kleinstädten und Landgemeinden (88 % zu 21 %, *p* < 0,001).

Für Allgemeinmediziner*innen steht eine Vielzahl an Informationsquellen zu digitalen Gesundheitstools bereit. Allerdings gibt nur knapp jede/r dritte Befragte an, häufig (3 %) oder gelegentlich (28 %) solche Quellen zu konsultieren (34 % selten). 35 % bekunden, sich nie über DiGA oder ähnliche Tools zu informieren. Mit 62 % ist dieser Anteil unter Landärzt*innen besonders hoch. Am häufigsten werden Informationen zu DiGA über Fachzeitschriften (24 %), Verbandszeitungen (22 %), den Austausch mit Kolleg*innen (21 %) sowie durch Internetrecherchen (20 %) bezogen (offene Frage, *n* = 2845). Als Online-Informationsquelle für Apps werden meist die Gesundheits-App-Plattform „HealthOn“ (15 %), das Zentrum der Telemedizin im Gesundheitswesen (12 %) und die Seiten des BfArM (11 %) genutzt.

Lediglich ein kleiner Teil traut sich zu, seriöse Gesundheits-Apps von schlechten unterscheiden zu können (22 %) oder das Angebot an verfügbaren Programmen grob zu überblicken (25 %). 22 % trauen sich zu, Patient*innen zu DiGA, Gesundheits-Apps und anderen digitalen Tools kompetent zu beraten. Während unter großstädtischen Ärzt*innen dieser Anteil 40 % beträgt, sind es unter landärztlichen Befragten 8 % (*p* < 0,001).

### Anwendungsbereitschaft und -erfahrung

Unabhängig davon, ob DiGA bereits eingesetzt wurden oder nicht, wurden die teilnehmenden Ärzt*innen zunächst danach gefragt, welche Grundvoraussetzungen eine DiGA erfüllen müsste, damit eine Verordnung infrage käme. Wie die Auswertung der offenen Frage (*n* = 3216) zeigt, ist den Befragten besonders wichtig, dass die DiGA übersichtlich bzw. leicht verständlich (64 %) und einfach bzw. intuitiv anwendbar ist (59 %). Sie soll personenbezogene Daten bestmöglich schützen (57 %), Möglichkeiten der Individualisierbarkeit bieten (54 %) sowie Patient*innen im Alltag auf spielerischem Weg zu mehr Gesundheitsbewusstsein motivieren (z. B. über Gamification-Elemente, 54 %). Ein erheblicher Teil des Samples betont als weitere Voraussetzung, dass Ärzt*innen zu dem entsprechenden Programm seriöse, belastbare Informationsquellen vorliegen müssen (46 %). Einige Befragte nennen eine dauerhafte Aufnahme ins DiGA-Verzeichnis als zwingende Voraussetzung für eine Verordnung (28 %).

Im Praxisalltag sprechen 31 % der Befragten bei Patient*innen häufig oder gelegentlich die Möglichkeit einer Unterstützung durch M‑Health-Tools wie DiGA oder Gesundheits-Apps zur Vorsorge oder Krankheitsbewältigung an (29 % selten). 17 % haben in der Vergangenheit häufig oder gelegentlich konkrete Apps zur Prävention, Lebensstiländerung und/oder Therapie empfohlen; bei weiteren 19 % kam dies selten vor.

Genuin bezogen auf DiGA haben 14 % aller Befragten bereits solche Anwendungen verschrieben. In dieser Gruppe fand die Verordnung bislang hauptsächlich in den Bereichen Prävention und Selbstkontrolle (75 %), Lifestyle (71 %) und Bewegungsförderung (62 %) statt. Eine Aufschlüsselung zeigt, dass 21 % der Ärzt*innen in Großstädten und 5 % der Ärzt*innen mit ländlichem Praxissitz DiGA bislang verordnet haben (*p* < 0,001). Weitere 13 % aller Ärzt*innen in der Gesamtstichprobe geben an, eine Verschreibung von DiGA für die nächste Zeit vorzuhaben, wobei diese ebenfalls auf die bereits genannten Bereiche abzielt. Für 52 % ist eine Verordnung grundsätzlich denkbar (21 % kommt grundsätzlich nicht infrage).

Nach ihrer grundsätzlichen Erfahrung gefragt, geben 83 % der Ärzt*innen mit DiGA-Erfahrung an, die verordneten Anwendungen hätten sich insgesamt als sehr nützlich (32 %) oder eher nützlich (51 %) erwiesen. Positive Effekte in Bezug auf Gesundheitsvorsorge und/oder Genesung wurden verbreitet beobachtet. Diese betreffen insbesondere Aspekte wie verbesserte Therapietreue und das Selbstmanagement bei chronischen Erkrankungen, die Steigerung der Mobilität oder eine feststellbare Gewichtsreduktion (Tab. 3). Mit Blick auf beobachtete Mehrwerte fällt das Urteil für DiGA in den Anwendungsbereichen Prävention und Selbstkontrolle, gesundheitsorientierter Lifestyle und Bewegungsförderung am besten aus.

**Table 3:** 

**Frage:** Hier stehen positive Effekte für den Gesundheitszustand. Was davon haben Sie bereits als Ergebnis einer DiGA-Nutzung Ihrer Patient*innen beobachtet? (Mehrfachangabe möglich)	Gesamtzustimmung (%)
Steigerung der Compliance (z. B. Medikamenteneinnahme, Therapieadhärenz)	95
Steigerung der Mobilität	94
Verbesserung von Gesundheitsbewusstsein und Aufklärung	93
Verbesserung des Selbstmanagements (z. B. bei chronischen Erkrankungen)	91
Gewichtsreduktion (z. B. Body-Mass-Index, Bauchumfang, Taillenumfang)	8
Verringern von Komplikationen (z. B. Hypoglykämie)	45
Rückgang von psychischen Problemen oder Begleiterscheinungen (z. B. Depression)	33
Abwehr von Folgeschäden (z. B. diabetisches Fußsyndrom, koronare Herzkrankheit)	21
Stabile Abnahme des Blutzuckers (HbA1c-Wert)	20
Rückgang des metabolischen Syndroms	20
Vermeiden von steigernden Therapieoptionen (z. B. Insulintherapie)	15
Vollständige Heilung	8

### Wahrgenommene Optimierungspotenziale

Um DiGA für die Anwendung im (haus-)ärztlichen Versorgungsgeschehen zugänglicher und damit attraktiver zu machen, umreißen die Ärzt*innen mit Nutzungserfahrung im Zuge einer offenen Frage unterschiedliche Schwerpunkte (Tab. 4). Trotz einer vergleichsweise hohen Zufriedenheit mit bereits verwendeten DiGA wird Nachsteuerungspotenzial bei der Nutzerführung und Nutzerfreundlichkeit sowie der systematischen Weiterbildung von Ärzt*innen und dem Ausbau von Interaktivität und spielerischen Elementen (Gamification) gesehen. Ebenfalls werden Aspekte der Honorierung, Informations‑, Daten- und Rechtssicherheit angesprochen. Zahlreichen Ärzt*innen fehlt es an zuverlässigen und fundierten Informationsmöglichkeiten über DiGA. Das DiGA-Verzeichnis wird oftmals kritisch gesehen – als nicht detailliert genug und mitunter zu nah an den Herstellerangaben abgefasst. Teilweise wird dies auch mit einer grundsätzlichen Kritik an der Berechtigung des „Fast-Track-Verfahrens“ verbunden. Als mögliche Informationsplattform mit einem DiGA-Schwerpunkt schlagen verschiedene Befragte das Nationale Gesundheitsportal (www.gesund.bund.de) vor, ggf. mit einer Sektion speziell für Ärzt*innen. Wichtig ist Ärzt*innen, dass DiGA in ihrer Funktionalität nicht einseitig auf nur wenige Vitalparameter fixiert sind und somit bei Patient*innen das Gefühl erzeugen, sich ständig mit diesen Parametern befassen zu müssen. Hier befürchtet ein Teil der Befragten, dass aufgrund einer solchen Fixierung Fehldeutungen bei Patient*innen entstehen oder Gesundheitsängste bzw. psychosomatische Beschwerden begünstigt werden könnten. Viele Hausärzt*innen äußern den Wunsch, dass die Krankenkassen mit Blick auf den DiGA-Einsatz stärker beratend und unterstützend an Patient*innen herantreten. Schon heute können bei entsprechender Indikation DiGA z. T. ohne explizite ärztliche Verordnung über die Krankenkasse bezogen werden [27].

**Table 4:** 

**Frage:** *Wenn Sie Ihre bisherige Erfahrung mit DiGA nehmen: Was sollte weiter rund um DiGA verbessert werden, was würden Sie sich wünschen?*	Gesamtzustimmung (%)
(Weitere) Optimierung der Usability von DiGA, insbesondere mit Blick auf eine einfache, intuitive und zielgruppenkonforme Nutzung (um z. B. Fehlmessungen oder fehlerhafter Anwendung vorzubeugen)	59
Die Gebührenordnung sieht eine angemessene Honorierung von ärztlichen Leistungen und des Mehraufwands vor, die im Zusammenhang mit DiGA erbracht werden	58
Systematische Weiterbildung von Ärzt*innen hinsichtlich des Einsatzes von DiGA in der (hausärztlichen) Patient*innenversorgung, u. a. durch Schaffung eines ausreichenden Angebots an CME-zertifizierten Fortbildungen	56
Neutrale und zuverlässige Informationsplattform mit Schwerpunkt auf DiGA, idealerweise staatlich administriert (Vorschlag u. a.: Nationales Gesundheitsportal)	52
Mehr Gamification-Elemente, stärkerer spielerischer und interaktiver Ansatz für Patient*innenführung	49
(Weitere) Verbesserung der Informations- und Datensicherheit (Schaffung von mehr verbindlichen und einheitlichen Datenschutzstandards für Hersteller)	47
Haftungsrisiken für Ärzt*innen klar ausschließen, wenn z. B. ein Behandlungsfehler aufgrund einer fehlerhaften DiGA entsteht (Verantwortung und Haftung soll nicht bei Leistungserbringern oder Patient*innen liegen)	44
DiGA sollten gezielt so konzipiert werden, dass sie nicht zum Aufkommen von Gesundheitsängsten führen (z. B. Ausschluss von Fehlinterpretation der Patient*innen oder eine Fokussierung auf einzelne Vitalparameter)	44
Verstärkte Beratung und Unterstützung von Patient*innen durch die Krankenkassen mit Blick auf DiGA	40
Hausärzt*innen stärker von Sinn und Nutzen einer (umfassenden) Digitalisierung des Gesundheitswesens überzeugen, um die Bereitschaft einer Integration in Versorgungsprozesse zu erhöhen	33
Streichung einer vorübergehenden DiGA-Zulassung via „Fast-Track-Verfahren“ (Verschärfung des Evaluationsverfahrens)	31
Technische Aspekte der Integration von DiGA in den Praxiskontext, z. B. kostenneutrale und funktionale Anbindung in die Praxissoftware	25
Versicherte aller Krankenkassen erhalten Prämien oder Bonusprogramme, wenn sie regelmäßig bestimmte DiGA nutzen und ihre Daten an die Krankenkasse weiterleiten	9

*CME* Continuing Medical Education

76 % der befragten Ärzt*innen sind prinzipiell bereit, DiGA deutlich stärker (18 %) oder etwas stärker (58 %) als bislang einzusetzen, soweit die von ihnen gewünschten Verbesserungen umgesetzt würden. 24 % sprechen sich dagegen aus.

## Diskussion

### Zusammenfassung und Befunde anderer Studien

Vorangegangene Studien haben gezeigt, dass niedergelassene und Allgemeinärzt*innen Gesundheits-Apps mit positiven Potenzialen in Verbindung bringen, allerdings aufgrund von beträchtlichen Bedenken bei Sicherheitsfragen, Zuverlässigkeit und Anwendungsfreundlichkeit bislang kaum bereit waren, M‑Health-Tools in die Patient*innenversorgung einzubinden [18, 20, 21]. Hinzu kommt ein hohes Maß an Unsicherheit, aus einem großen, dynamischen App-Markt passende Anwendungen für Patient*innen auszuwählen. Das DVG folgt dem Bestreben, mithilfe klarer Qualitätsstandards die Grundlagen für eine Implementierung von DiGA in die Versorgung zu schaffen [1, 3].

Tatsächlich belegen die Ergebnisse der vorliegenden Befragung im Abgleich mit früheren Erhebungen, dass das Image und die Akzeptanz von DiGA unter Hausärzt*innen merklich positiver ausfallen als im Fall gewöhnlicher Gesundheits-Apps [19–22]. Aufgrund der notwendigen Prüfung für die Aufnahme ins DiGA-Verzeichnis und des rechtlichen Rahmenwerks zeigen Hausärzt*innen insgesamt größeres Vertrauen, dass es sich bei DiGA um seriöse, vergleichsweise sichere und potenziell wirksame Programme handelt – ein Befund, den auch eine BARMER-Umfrage andeutet [28]. Eine Befragung der Stiftung Gesundheit von 569 ambulanten Ärzt*innen und Psychotherapeut*innen hat trotz grundlegend abweichender Befragtenzielgruppen ebenfalls gezeigt, dass mehrheitlich Vertrauen in den Einsatz von medizinischen Apps auf Rezept besteht [29]. Obwohl die Zahl der Ärzt*innen mit Verordnungserfahrung bislang noch vergleichsweise gering ist, ist der Einsatz von DiGA für zahlreiche Befragte erwägenswert oder wird angestrebt.

Ähnlich wie im Fall von qualitativ hochwertigen Gesundheits-Apps werden DiGA v. a. bei der Unterstützung von Prävention, Selbstkontrolle und Veränderungen des Lebensstils als besonders sinnvoll erachtet. Auch diesbezüglich finden sich deutliche Parallelen in der allgemeinen Befragung der Stiftung Gesundheit [29]. Innerhalb einer durchgeführten Faktorenanalyse bezog sich der größte Cluster unter den Befragten auf wahrgenommene Vorteile von DiGA für die Gesundheitsförderung, Therapietreue und Motivation; Befragte in einem zweiten Cluster bezogen sich v. a. auf Effektivitäts- und Effizienzvorteile für die Arzt-Patient-Vernetzung.

Analog gilt diese positive Einschätzung für praktische Erfahrungen mit DiGA: Eine deutliche Mehrheit der Befragten, die solche Programme bereits verschrieben haben, hat günstige Versorgungseffekte beobachtet. Im Zusammenhang mit der Anwendungsbereitschaft fällt auf, dass jüngere Hausärzt*innen und solche in urbanen Räumen DiGA erheblich aufgeschlossener und nutzungsaffiner begegnen. Dies ist im betrachteten thematischen Kontext ein durchaus verbreiteter Befund und korrespondiert mit dem Umstand, dass jüngere Ärzt*innen eine geringere Hürde beim Aufgreifen neuer digitaler Technologien verspüren [30]. In urbanen Umgebungen – in denen Ärzt*innen bereits im Durchschnitt jünger sind als in ländlichen Umgebungen – sind Rahmen- und Begleitfaktoren hierfür oftmals günstiger (z. B. Anbindung an digitale Infrastruktur, Arbeitsteiligkeit, Vorhandensein digitalaffiner Ärzt*innennetzwerke).

Trotz der prinzipiell positiven Beurteilung von DiGA und deren Nutzungsmöglichkeiten ist die Bereitschaft, diese breitflächig und konsequent für die Patient*innenversorgung aufzugreifen, aktuell noch begrenzt [15–17, 20, 29]. Zum einen führen mangelnde Vorerfahrungen mit M‑Health-Programmen dazu, dass sich die meisten Befragten derzeit nicht zutrauen, Patient*innen kompetent an DiGA heranzuführen und die Anwendung zu begleiten [31, 32]. Zum anderen fehlen belastbare Informationsquellen, sodass Allgemeinmediziner*innen einen ausgeprägten Bedarf an neutralen Recherchequellen mit einem Schwerpunkt auf Gesundheits-Apps artikulieren. Das DiGA-Verzeichnis wird für die qualifizierte Übersicht und Auswahl geeigneter Anwendungen als verbesserungsbedürftig bewertet. Mehrere vorangegangene Untersuchungen haben aufgezeigt, dass Hausärzt*innen die Transparenz und Verlässlichkeit von derzeit zur Verfügung stehenden Informationsquellen als nicht ausreichend für eine Patient*innenunterstützung mit DiGA bzw. Gesundheits-Apps erachten [13, 17–22, 28].

Als mögliche Informationsplattform mit einem solchen Schwerpunkt schlagen verschiedene Befragte das Nationale Gesundheitsportal (www.gesund.bund.de) vor, ggf. mit einer Sektion speziell für Ärzt*innen. Ein ähnliches Resultat hat bereits eine von den Autoren durchgeführte Hausärzt*innenbefragung zu besagtem Portal ergeben [33]. Fachgesellschaften und deren Organe könnten mithilfe eigener Informationsangebote unterstützen und Versorgungsergebnisse von DiGA diskutieren. Ferner wünschen sich die Befragten ein breites Angebot an CME-zertifizierten^1^, professionellen Fort- und Weiterbildungen, die Hausärzt*innen die Möglichkeiten und Rahmenbedingungen einer Integration von DiGA ins Versorgungsgeschehen näherbringen [23, 34]. Dass vielen Allgemeinmediziner*innen die Grundlagen des DVG (z. B. zu Datensicherheit, Honorierung oder Rechtsfragen) nicht geläufig sind, spiegelt sich ebenfalls in den Ergebnissen. Aus Sicht der Befragten wäre zudem von großer Bedeutung, dass Krankenkassen Patient*innen in Bezug auf eine DiGA-Nutzung konsequent und proaktiv beraten und Ärzt*innen diese Aufgabe nicht allein überlassen bleibt.

Mit Blick auf eine weitere Optimierung heben Ärzt*innen mit DiGA-Erfahrung auf die Stärkung von motivationsförderlicher Anwenderfreundlichkeit ab, sodass eine unkomplizierte Bedienbarkeit auch für nicht digitalaffine oder kognitiv eingeschränkte Patient*innen gewährleistet ist [24, 35]. Krisam und Preis [36] raten bei der (Weiter‑)Entwicklung von DiGA explizit zu einem Ausbau des spielerischen Potenzials. Durch die Implementierung spielerischer Elemente (Gamification) und eine intuitive Anwendbarkeit (User Experience) lassen sich Verhaltensänderungen niederschwellig und nachhaltig anregen.

### Stärken und Schwächen

Die Befragung hat auf quantitativen und qualitativen Vorstudien aufgebaut und war daher auf die hausärztliche Perspektive zugeschnitten. Mehrere offene Fragen ermöglichten die Erfassung nicht standardisierter Informationen. Zudem erzielte die Befragung einen vergleichsweise hohen Rücklauf, sodass eine Stichprobe erzielt wurde, die hinsichtlich wichtiger Merkmale und Haltungen in Bezug auf DiGA und deren Anwendungspotenzial breit gestreut ist.

Nichtsdestotrotz kann die Studie nicht im strengen Sinne, sondern – durch einen Vergleich mit KV-Daten – höchstens angenähert einen repräsentativen Anspruch erheben (begrenzte Fallzahl, regionale Rekrutierung). Zudem kann nicht ausgeschlossen werden, dass Ärzt*innen mit thematischem Interesse (Digitalisierung, Gesundheits-Apps) in stärkerem Maße teilgenommen haben. Darauf weist z. B. ein im Vergleich zu anderen Studien relativ hoher Anteil von Hausärzt*innen mit bestehender DiGA-Erfahrung hin [29]. Dennoch zeigt die gewonnene Stichprobe, dass die Befragten hinsichtlich wichtiger Merkmale und Haltungen in Bezug auf DiGA und deren Anwendungspotenzial breit gestreut sind.

Ein zusätzlicher Kritikpunkt gilt der im Fragebogen verwendeten, nicht einheitlichen Skalierung, z. B. wurden verschiedene Skalenstufen verwendet oder ergänzende Kategorien wie „unentschieden“ oder „schwer zu sagen“. Der Grund hierfür lag im Bestreben der Autoren, angesichts der zeitkritischen Zielgruppe eine möglichst einfache Ausfüllbarkeit des Instruments zu gewährleisten.

## Fazit

DiGA werden von Hausärzt*innen im Hinblick auf ihr Versorgungspotenzial überwiegend positiv wahrgenommen und im Vergleich zu gewöhnlichen Gesundheits-Apps als sicherer und zuverlässiger bewertet. Ärzt*innen mit Nutzungserfahrung berichten vielfach von positiven Versorgungseffekten aufgrund der DiGA-Intervention. Für zahlreiche Allgemeinmediziner*innen ist ein Einsatz von DiGA in der Zukunft erwägenswert. Damit bieten sich günstige Voraussetzungen für eine Implementierung in der Primärversorgung. Dennoch wird das Nutzungspotenzial von DiGA bislang noch nicht ausgeschöpft.

Um DiGA flächendeckend in der hausärztlichen Praxis zu verankern, kommt es darauf an, Allgemeinmediziner*innen über die Grundlagen des DVG zu informieren und Bedenken bzw. Wünsche gezielt zu adressieren, um die Akzeptanz hausärztlicher Versorger*innen mittel- und langfristig zu erhöhen. Zentral erscheinen flächendeckende Schulungen, die über Rahmenbedingungen, Nutzen und Limitationen des DiGA-Einsatzes aufklären und Strategien aufzeigen, wie sich digitale Tools systematisch einbinden lassen. Auch besteht Bedarf an verstärkter Orientierung und Überblick, um sicher einschätzen zu können, welche DiGA für welches Anwendungsgebiet sinnvoll und was beim Einsatz zu beachten ist.

## Supplementary Information





## Footnotes

1 CME (Continuing Medical Education): kontinuierliche berufsbegleitende Fortbildung für Ärzt*innen, die in der Medizin tätig sind; CME-Zertifikat: Fortbildungszertifikat der zuständigen Ärztekammer.
