# Supplementary material for: What potential and added value do DiGA offer for primary care?—Results of a survey of general practitioners in Germany
Source: Bundesgesundheitsblatt Gesundheitsforschung Gesundheitsschutz. 2022 Oct 21;65(12):1334–43. [Article in German] doi: 10.1007/s00103-022-03608-w (PMC9722862; doi:10.1007/s00103-022-03608-w)
Supplement: Supplementary file 1 [file 103_2022_3608_MOESM1_ESM.pdf]

1. Seit 2020 gibt es für Ärzt*innen die Möglichkeit, Patient*innen digitale Gesundheitsanwendungen, kurz: DiGA, auf Rezept zu verordnen. Als verschreibungsfähige mobile Apps sollen DiGA dabei helfen, Erkrankungen besser zu erkennen und zu behandeln oder die Gesundheitsvorsorge unterstützen. Im Gegensatz zu gewöhnlichen Gesundheits-Apps, die man ohne Rezept bekommt, gelten DiGA als Medizinprodukte. DiGA werden in einem Verfahren des Bundesinstituts für Arzneimittel und Medizinprodukte (BfArM) eingehend geprüft und bei positivem Ergebnis in ein Verzeichnis erstattungsfähiger Programme aufgenommen.

War Ihnen bekannt, dass es solche DiGA gibt, oder hören Sie dies zum ersten Mal?

◯ War mir bekannt ◯ Höre ich zum ersten Mal

2. Unabhängig davon, ob Ihnen bekannt war, dass es DiGA gibt: Halten Sie solche verschreibungsfähige medizinische Apps grundsätzlich für eine gute Sache, oder stehen Sie dem eher skeptisch gegenüber?

◯ Gute Sache ◯ Eher Skeptisch ◯ Unentschieden

3. Wie eingangs erwähnt, werden DiGA in einem systematischen Verfahren des Bundesinstituts für Arzneimittel und Medizinprodukte (BfArM) eingehend geprüft. Dies bedeutet, dass DiGA-Hersteller einen Antrag auf Zulassung stellen müssen und als Voraussetzung für eine Aufnahme in das DiGA-Verzeichnis eine Reihe von Kriterien erfüllt sein müssen, darunter Standards zu Datenschutz und Informationssicherheit, Qualität medizinischer Inhalte, Nutzerfreundlichkeit und Robustheit der Anwendung sowie Patient*innensicherheit. Nutzen und Mehrwert der Anwendung (Versorgungs-effekt) müssen ausreichend belegt werden. (Z.B. kann ein Rückgang von Schmerzen ein positiver Versorgungseffekt sein, aber auch ein größeres Wissen zur Erkrankung, das zu einem besseren Umgang damit führt.)

Unabhängig davon, ob Sie schon persönliche Erfahrungen mit DiGA gemacht haben oder nicht: Gehen Sie davon aus, dass solche vom BfArM geprüften digitale Gesundheitsanwendungen verlässliche Programme sind, die man als Ärzt*in ggf. Patient*innen empfehlen und verschreiben kann? (Gemeint ist Ihr grundsätzliches Vertrauen, dass durch ein solches Prüfverfahren eine Auswahl an seriösen Programmen geschaffen wird, die bedenkenlos empfohlen werden können.)

Ich gehe… ◯ stark davon aus ◯ eher davon aus ◯ eher nicht davon aus ◯ überhaupt nicht davon aus ◯ Unentschieden

4. Gehen Sie davon aus, dass es bei der Verschreibung von DiGA und deren Nutzung im Praxiskontext ausreichend Rechtssicherheit für Ärzt*innen gibt (mit Blick auf Fragen von Risiken und Haftungsfragen)?

Ich gehe… ◯ stark davon aus ◯ eher davon aus ◯ eher nicht davon aus ◯ überhaupt nicht davon aus ◯ Unentschieden

5. Welchen Beitrag können DiGA Ihrer Meinung oder auch Erfahrung nach zur Gesundheitsvorsorge und/oder Genesung von Patient*innen – die richtige Anwendung vorausgesetzt – leisten?
Ist der Beitrag, den DiGA insgesamt Ihrer Ansicht nach leisten können…

◯ Sehr groß ◯ Eher groß ◯ Eher gering ◯ Leisten keinen Beitrag

6. In welchen der folgenden Anwendungsbereiche sind DiGA Ihrer Ansicht nach sinnvoll und können eine positive Wirkung bei Patient*innen entfalten?

	Sehr sinnvoll	Eher sinnvoll	Weniger bzw. gar nicht sinnvoll	Schwer zu sagen
Prävention, z.B. Selbstkontrolle von Risikofaktoren (Gewicht, Blutdruck, Blutzucker etc.) oder Gesundheitsdaten (gelaufene Schritte, Trinkmenge, Ernährung etc.)	◯	◯	◯	◯
Monitoring und Therapie chronischer Erkrankungen (Diabetes, Asthma, Schmerz etc.)	◯	◯	◯	◯
Lifestyle, Einhaltung eines gesundheitsförderlichen Lebensstils (Ernährung, Rauchen, Psyche etc.)	◯	◯	◯	◯
Unterstützung von physischen Maßnahmen (Bewegungsübungen in Eigenregie etc.)	◯	◯	◯	◯
Erinnerung an die regelmäßige Einnahme von Medikamenten, Erinnerung an Impf- und Vorsorgetermine	◯	◯	◯	◯
Sonstiges, und zwar: ________________________________	◯	◯	◯	◯

7. Welchen der folgenden Aussagen stimmen Sie tendenziell zu? Bitte kreuzen Sie jeweils an.

◯ DiGA entlasten Ärzt*innen und Pflegekräfte, weil diese sich nicht mehr um die Erfassung von Vitaldaten und Messwerten kümmern müssen

◯ Die Anwendung von DiGA ist für viele Patient*innen zu kompliziert, wodurch fehlerhafte Gesundheitsdaten gesammelt werden oder im Extremfall Therapien fehlschlagen

◯ DiGA stärken die Motivation von Patient*innen, etwas für ihre Gesundheit zu tun

◯ DiGA geben Patient*innen das Gefühl, stärker Kontrolle über ihre Gesundheit und Gesunderhaltung zu haben (Empowerment)

◯ Ich glaube nicht, dass DiGA personenbezogene Daten ausreichend schützen

◯ Durch die zusätzlichen Informationen, die von DiGA erfasst werden, können Patient*innen individueller und effektiver behandelt werden

◯ Die Nutzung von DiGA ist für Patient*innen und Ärzt*innen zu zeitaufwändig

◯ DiGA erleichtern die Kommunikation zwischen Arzt und Patient

◯ DiGA erzeugen eine Flut von Daten, die einer effektiven Behandlung nicht zweckdienlich sind

◯ Durch DiGA lassen sich Arztkontakte zeitlich effektiver gestalten

◯ DiGA stärken die Compliance von Patient*innen

◯ Durch DiGA wird das Arzt-Patient-Verhältnis unpersönlicher

◯ DiGA tragen dazu bei, dass Patient*innen besser über Gesundheits- und Krankheitsthemen aufgeklärt sind

◯ Durch den Einsatz von DiGA beim Patienten wird der Arzt stärker belastet als entlastet, weil zusätzliche Aufgaben für ihn anfallen

◯ Krankheiten und Krankheitsrisiken können aufgrund der Nutzung von DiGA schneller erkannt bzw. diagnostiziert werden

◯ DiGA führen dazu, dass Patient*innen ihre Diagnose und Therapie in die eigene Hand nehmen

◯ Mit DiGA lassen sich neue Patient*innengruppen erreichen

8. Gehen Sie davon aus, dass DiGA die Digitalisierung von Gesundheitswesen und Arztpraxen in positiver und sinnvoller Weise voranbringen werden, oder gehen Sie nicht davon aus?

Ich gehe… ◯ stark davon aus ◯ eher davon aus ◯ eher nicht davon aus ◯ überhaupt nicht davon aus ◯ Schwer zu sagen

9. Haben Sie bereits DiGA bei Patient*innen verschrieben, oder haben Sie dies in kommender Zeit vor, können Sie sich dies grundsätzlich vorstellen, oder kommt eine Verschreibung von DiGA grundsätzlich nicht für Sie in Frage?

◯ Bereits verschrieben ◯ Habe ich vor => Bitte direkt übergehen zu Frage 17

◯ Kann ich mir vorstellen => Bitte direkt übergehen zu Frage 17

◯ Kommt grundsätzlich nicht in Frage => Bitte direkt übergehen zu Frage 18

10. Sie haben angegeben, dass Sie DiGA bereits eingesetzt bzw. Patient*innen verschrieben haben. Hierzu möchten wir Ihnen im Folgenden einige Fragen zu Ihren bisherigen Erfahrungen stellen.

Wie oft ist es bereits vorgekommen, dass Sie im Gespräch mit Patient*innen die Möglichkeit einer Unterstützung durch Gesundheits-Apps oder DiGA zur Unterstützung der Vorsorge oder Krankheitsbewältigung von sich aus angesprochen haben?

◯ Häufig ◯ Gelegentlich ◯ Selten ◯ Nie ◯ Schwer zu sagen, weiß nicht

11. Wie häufig ist es bereits vorgekommen, dass Sie Patient*innen die Nutzung von bestimmten Gesundheits-Apps zur Prävention und/oder Therapie empfohlen haben? (Gemeint sind nicht nur DiGA, sondern alle Arten von Gesundheits-Apps.)

◯ Häufig ◯ Gelegentlich ◯ Selten ◯ Nie ◯ Schwer zu sagen, weiß nicht

12. In welchen Anwendungsbereichen haben Sie DiGA bei Ihren Patient*innen eingesetzt? Bitte geben Sie für den oder die jeweiligen Anwendungsbereich auch an, wie Sie den Nutzen der von Ihnen eingesetzten DiGA insgesamt beurteilen.

		Falls eingesetzt,…		
	Eingesetzt	Sehr nützlich	Eher nützlich	Weniger bzw. gar nicht nützlich
Prävention, z.B. Selbstkontrolle von Risikofaktoren (Gewicht, Blutdruck, Blutzucker etc.) oder Gesundheitsdaten (gelaufene Schritte, Trinkmenge, Ernährung etc.)	◯	◯	◯	◯
Monitoring und Therapie chronischer Erkrankungen (Diabetes, Asthma, Schmerz etc.)	◯	◯	◯	◯
Lifestyle, Einhaltung eines				

gesundheitsförderlichen Lebensstils (Ernährung, Rauchen, Psyche etc.)	◯	◯	◯	◯
Unterstützung von physischen Maßnahmen (Bewegungsübungen in Eigenregie etc.)	◯	◯	◯	◯
Erinnerung an die regelmäßige Einnahme von Medikamenten, Erinnerung an Impf- und Vorsorgetermine	◯	◯	◯	◯
Sonstiges, und zwar: ______________________________	◯	◯	◯	◯

13. Falls Sie dies noch wissen: Welche DiGA haben Sie Patient*innen vor allem verschrieben? Bitte nennen Sie nach Möglichkeit 1-3 Beispiele.

____

____

14. Hier stehen positive Effekte für den Gesundheitszustand. Was davon haben Sie bereits als Ergebnis einer DiGA-Nutzung Ihrer Patient*innen beobachtet? (Mehrfachnennung möglich)

◯ Verbesserung von Gesundheitsbewusstsein und Aufklärung
◯ Steigerung der Compliance (z.B. Medikamenteneinnahme, Therapieadhärenz)
◯ Verbesserung des Selbstmanagements (z.B. bei chronischen Erkrankungen)
◯ Gewichtsreduktion (z.B. BMI, Bauchumfang, Taillenumfang)
◯ Stabile Abnahme des Blutzuckers (HbA1c)
◯ Rückgang des metabolischen Syndroms
◯ Rückgang von psychischen Problemen oder Begleiterscheinungen (z.B. Depression)
◯ Abwehr von Folgeschäden (z.B. diabetisches Fußsyndrom, KHK)
◯ Verringern von Komplikationen (z.B. Hypoglykämie)
◯ Vermeiden von steigernden Therapieoptionen (z.B. Insulintherapie)
◯ Steigerung der Mobilität
◯ Vollständige Heilung
◯ Sonstiges: ____

15. Wenn Sie Ihre bisherige Erfahrung mit DiGA nehmen: Was sollte weiter rund um DiGA verbessert werden, was würden Sie sich wünschen? (Bitte geben Sie hier Ihre kritische Rückmeldung ab.)

____

____

____

16. Einmal angenommen, die von Ihnen angegebenen Punkte würden umgesetzt: Wären Sie unter diesen Voraussetzungen bereit, DiGA in der Patientenversorgung stärker als bislang zu berücksichtigen?

◯ Ja, deutlich stärker ◯ Ja, etwas stärker ◯ Nein ◯ Habe keine weiteren Wünsche angegeben

⇨ Bitte gehen Sie direkt über zu Frage 18

17. Sie haben angegeben, dass Sie DiGA bislang noch nicht eingesetzt haben. In welchen der folgenden Anwendungsbereiche wäre es für Sie vorstellbar, DiGA künftig einzusetzen?

	Sehr gut vorstellbar	Eher gut vorstellbar	Weniger bzw. gar nicht gut vorstellbar
Prävention, z.B. Selbstkontrolle von Risikofaktoren (Gewicht, Blutdruck, Blutzucker etc.) oder Gesundheitsdaten (gelaufene Schritte, Trinkmenge, Ernährung etc.)	◯	◯	◯
Monitoring und Therapie chronischer Erkrankungen (Diabetes, Asthma, Schmerz etc.)	◯	◯	◯
Lifestyle, Einhaltung eines gesundheitsförderlichen Lebensstils (Ernährung, Rauchen, Psyche etc.)	◯	◯	◯
Unterstützung von physischen Maßnahmen (Bewegungsübungen in Eigenregie etc.)	◯	◯	◯
Erinnerung an die regelmäßige Einnahme von Medikamenten, Erinnerung an Impf- und Vorsorgetermine	◯	◯	◯

18. Was muss bzw. müsste eine DiGA Ihrer Ansicht nach erfüllen, damit Sie diese Patient*innen empfehlen? Was ist Ihnen besonders wichtig? Bitte nennen Sie 1-5 Kriterien.

____

____

19. Was schätzen Sie: Wie viele Ihrer Patient*innen nutzen wenigstens zeitweise Gesundheits-Apps und/oder andere digitale Tools zur gesundheitlichen Vorsorge bzw. zum Krankheitsmanagement?

◯ Weniger als 5% ◯ 5-10% ◯ 10-15% ◯ 15-20% ◯ 20-25% ◯ Mehr als 25% ◯ Weiß nicht

20. Wenn Sie Ihre Patientenschaft insgesamt betrachten: Was schätzen Sie, wie viele Patient*innen grundsätzlich daran interessiert wären, DiGA zur Vorsorge oder Krankheitsbewältigung zu nutzen?

ca. ____________ %

21. Wie häufig ist es bereits vorgekommen, dass Sie von Patient*innen auf DIGA angesprochen wurden?

◯ Häufig ◯ Gelegentlich ◯ Selten ◯ Nie

22. Ist es Ihrer Ansicht nach in Ordnung, wenn man sich als Arzt bei der Therapieplanung prinzipiell auf vom Patienten mittels Gesundheits-Apps erfasste Daten verlässt, oder sind solche Daten Ihrer Ansicht nach für die Therapieplanung kritisch zu sehen?

◯ In Ordnung ◯ Kritisch zu sehen ◯ Schwer zu sagen

23. Wie häufig kommt es vor, dass Sie sich über DiGA, Gesundheits-Apps und/oder andere digitale Tools informieren?

◯ Häufig ◯ Gelegentlich ◯ Selten ◯ Nie => Bitte übergehen zu Frage 25

24. Über welche Quellen (z.B. Internetseiten) informieren Sie sich schon mal darüber?

____

____

25. Wie schätzen Sie Ihr Wissen bzw. Ihre Kompetenz ein, wenn es darum geht, das Angebot an verfügbaren Gesundheits-Apps und anderen digitalen Tools zu überblicken?

◯ Sehr gut ◯ Eher gut ◯ Weniger gut ◯ Gar nicht gut

26. Wie schätzen Sie Ihr Wissen bzw. Ihre Kompetenz ein, wenn es darum geht, gute von schlechten Gesundheits-Apps zu unterscheiden?

◯ Sehr gut ◯ Eher gut ◯ Weniger gut ◯ Gar nicht gut

27. Wie schätzen Sie Ihr Wissen bzw. Ihre Kompetenz ein, wenn es darum geht, Patient*innen zu Gesundheits-Apps und anderen digitalen Tools, etwa zur Prävention und/oder Therapie, zu beraten?

◯ Sehr gut ◯ Eher gut ◯ Weniger gut ◯ Gar nicht gut

Nun bräuchten wir noch einige statistische Angaben von Ihnen. Wie auch der Rest des Fragebogens werden diese Informationen natürlich streng vertraulich behandelt und anonymisiert.

Sind sind…

◯ Männlich ◯ Weiblich ◯ Divers

Ihr **Alter**: _____

Wo befindet sich Ihre Praxis? In einer Gemeinde/Stadt mit . . .

◯ mehr als 100.000 ◯ 20.000 bis 100.000 ◯ 5.000 bis 20.000 ◯ unter 5.000 Einwohnern

Welches Niederlassungsmodell beschreibt Ihre Praxis am besten?

◯ Einzelpraxis (Sie sind der/die einzige Arzt/Ärztin) ◯ Einzelpraxis mit angestellten Ärzten
◯ Gemeinschaftspraxis ◯ Medizinisches Versorgungszentrum ◯ Sonstiges

Wie viele Patient*innen behandelt Ihre Praxis im Quartal?

◯ 500 bis 750 ◯ 751 bis 1000 ◯ 1001 bis 1500 ◯ 1501 bis 2000 ◯ Mehr als 2000

Vielen Dank für Ihre Teilnahme!

Was möchten Sie uns noch mitteilen? Hier finden Sie Platz für Anregungen, Kommentare und Kritik.

____

____
